# Collaborative studies in toxicogenomics in rodent liver in JEMS·MMS; a useful application of principal component analysis on toxicogenomics

**DOI:** 10.1186/s41021-016-0041-0

**Published:** 2016-08-01

**Authors:** Chie Furihata, Takashi Watanabe, Takayoshi Suzuki, Shuichi Hamada, Madoka Nakajima

**Affiliations:** 1 School of Science and Engineering, Aoyama Gakuin University, Sagamihara, Kanagawa 252-5258 Japan; 2 Division of Molecular Target and Gene Therapy Products, National Institute of Health Sciences, Setagaya-ku, Tokyo, 158-8501 Japan; 3 Laboratory for Integrative Genomics, RIKEN Center for Integrative Genomics, RIKEN Yokohama Institute, 1-7-22 Suehiro-cho, Tsurumi-ku, Yokohama, 230-0045 Japan; 4 Nonclinical Research Center, Drug Development Service Segment, LSI Medience Corporation, Kamisu-shi, Ibaraki 314-0255 Japan; 5 Genetic Toxicology Group, Biosafety Research Center, Foods, Drugs, and Pesticides, Shioshinden 582-2, Fukude-cho, Iwata-gun, Shizuoka 437-1213 Japan; 6 Education and Research Department, University of Shizuoka, Shizuoka, 422-8526 Japan

**Keywords:** Toxicogenomics, Hepatocarcinogen, Rodent liver, DNA microarray, Quantitative real-time PCR, Principal component analysis, Gene network

## Abstract

Toxicogenomics is a rapidly developing discipline focused on the elucidation of the molecular and cellular effects of chemicals on biological systems. As a collaborative study group of Toxicogenomics/JEMS·MMS, we conducted studies on hepatocarcinogens in rodent liver in which 100 candidate marker genes were selected to discriminate genotoxic hepatocarcinogens from non-genotoxic hepatocarcinogens. Differential gene expression induced by 13 chemicals were examined using DNA microarray and quantitative real-time PCR (qPCR), including eight genotoxic hepatocarcinogens [*o*-aminoazotoluene, chrysene, dibenzo[*a,l*]pyrene, diethylnitrosamine (DEN), 7,12-dimethylbenz[*a*]anthracene, dimethylnitrosamine, dipropylnitrosamine and ethylnitrosourea (ENU)], four non-genotoxic hepatocarcinogens [carbon tetrachloride, di(2-ethylhexyl)phthalate (DEHP), phenobarbital and trichloroethylene] and a non-genotoxic non-hepatocarcinogen [ethanol]. Using qPCR, 30 key genes were extracted from mouse livers at 4 h and 28 days following dose-dependent gene expression alteration induced by DEN and ENU: the most significant changes in gene expression were observed at 4 h. Next, we selected key point times at 4 and 48 h from changes in time-dependent gene expression during the acute phase following administration of chrysene by qPCR. We successfully showed discrimination of eight genotoxic hepatocarcinogens [2-acetylaminofluorene, 2,4-diaminotoluene, diisopropanolnitrosamine, 4-dimethylaminoazobenzene, 4-(methylnitsosamino)-1-(3-pyridyl)-1-butanone, *N*-nitrosomorpholine, quinoline and urethane] from four non-genotoxic hepatocarcinogens [1,4-dichlorobenzene, dichlorodiphenyltrichloroethane, DEHP and furan] using qPCR and principal component analysis. Additionally, we successfully identified two rat genotoxic hepatocarcinogens [DEN and 2,6-dinitrotoluene] from a nongenotoxic-hepatocarcinogen [DEHP] and a non-genotoxic non-hepatocarcinogen [phenacetin] at 4 and 48 h. The subsequent gene pathway analysis by Ingenuity Pathway Analysis extracted the DNA damage response, resulting from the signal transduction of a p53-class mediator leading to the induction of apoptosis. The present review of these studies suggests that application of principal component analysis on the gene expression profile in rodent liver during the acute phase is useful to predict genotoxic hepatocarcinogens in comparison to non-genotoxic hepatocarcinogens and/or non-carcinogenic hepatotoxins.

## Background

Recently, a radical overhaul of toxicological test protocols has been proposed [1–4]. For example, Hartung wrote that after several productive decades, in which a patchwork of testing approaches was formed, fewer and fewer of the latest scientific development were incorporated [1]. Caiment et al. [4] wrote that one of the main challenges of toxicology is the accurate prediction of compound carcinogenicity. The default test model for assessing chemical carcinogenicity, the 2-year rodent cancer bioassay, is currently criticized because of its limited specificity. With increased societal attention and new legislation against animal testing, toxicologists urgently need an alternative to the current rodent bioassays for chemical cancer risk assessment. In the beginning of the 21st century, toxicogenomics approaches proposed to use global high-throughput technologies (transcriptomics) to study the toxic effect of compounds on a biological system.

For risk assessment purposes, there is a general agreement that the chemicals acting through genotoxic and non-genotoxic mechanisms of carcinogenesis should be distinguished [5]. Mathijs et al. hypothesized that genotoxic and non-genotoxic carcinogens induce distinct gene expression profiles, which may therefore be used to classify the mechanisms of compounds as either genotoxic carcinogens or non-genotoxic carcinogens [6]. DNA microarray, which is a powerful technology for characterizing gene expression on a genome-wide scale [7], developed toxicogenomics. Quantitative real-time PCR (qPCR) is the field standard for measuring gene expression and is the most sensitive technique for the detection and quantification of mRNA target [8].

In the present study, we summarize our collaborative studies in toxicogenomics. We first selected about 100 candidate marker genes to discriminate mouse genotoxic hepatocarcinogens from non-genotoxic hepatocarcinogens by DNA microarrays, which were next quantified by qPCR [9]. We extracted about 30 key genes from dose responses in gene expression [10] and selected key point times at the beginning and end of the acute phase (4 and 48 h) [11]. We successfully showed the discrimination of genotoxic and non-genotoxic hepatocarcinogens in mouse liver [12] and rat liver [13] by qPCR and the application of principal component analysis (PCA) at 4 and 48 h after administration of hepatocarcinogens. The subsequent gene pathway analysis by Ingenuity Pathway Analysis extracted the DNA damage response, resulting from signal transduction by a p53-class mediator leading to the induction of apoptosis. Application of PCA was useful to discriminate genotoxic hepatocarcinogens from non-genotoxic and/or non-genotoxic non-hepatocarcinogens on rodent liver.

### Selection of genes by DNA microarray and quantified by real-time PCR

In our preliminary study, we examined differential gene expression of 13 chemicals including eight genotoxic hepatocarcinogens [*o*-aminoazotoluene, chrysene, dibenzo[*a,l*]pyrene, DEN, 7,12-dimethylbenz[*a*]anthracene, dimethylnitrosamine, dipropylnitrosamine, and ENU], four non-genotoxic hepatocarcinogens [carbon tetrachloride, DEHP, phenobarbital, and trichloroethylene], and a non-genotoxic non-hepatocarcinogen (for mouse) [ethanol] using DNA microarray (Affymetrix GeneChip Mu74A V2 and in-house microarray) in mouse liver at 4 h and up to 28 days following a single intraperitoneal administration to groups of five 9-week-old B6C3F1 male mice. The cDNA was prepared with total RNA combined from pooled livers. After preliminary DNA microarray data were generated, results were confirmed by qPCR. We identified about 100 candidate genes to discriminate the genotoxic hepatocarcinogens from the non-genotoxic hepatocarcinogens. The results were published in part [9] and registered to the GEO database (GEO accession GSE33248). The changes in gene expression at 4 h were much greater than at 20 h, 14 days, and 28 days. We used qPCR in continual studies.

### Dose-dependent alterations in gene expression at 4 h and 28 days

**Fig. 1:**
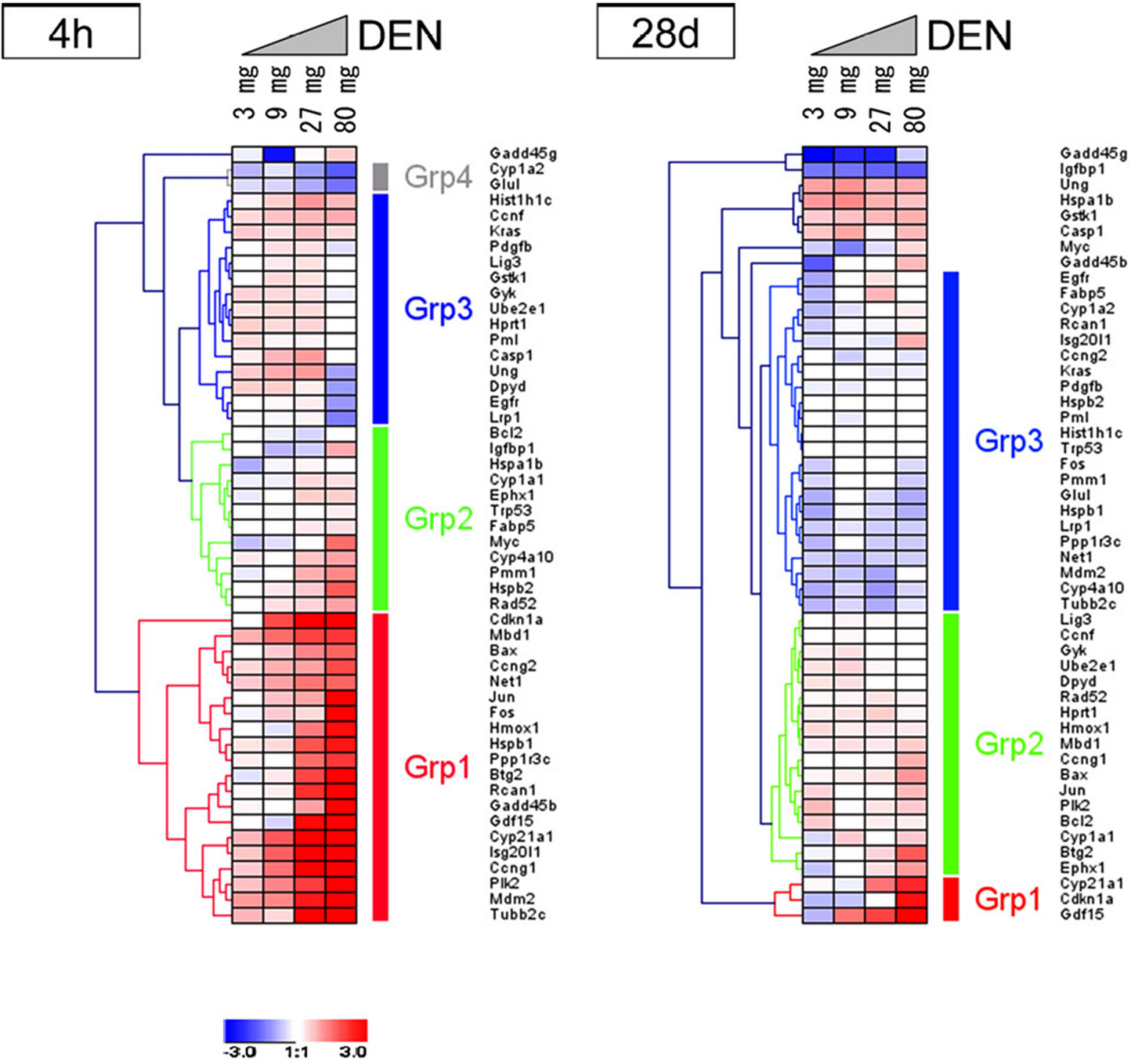
Cluster analysis of gene expression after DEN treatment. The expression of 50 genes was clustered by hierarchical clustering after DEN treatment. Results of 4 h and 28 days were analyzed separately. The color displays show the log_2_ (expression ratio) as (1) red when the treatment sample is up-regulated relative to the control (vehicle) sample, (2) blue when the treatment sample is down-regulated relative to the control sample and (3) white when the log_2_ (expression ratio) is close to zero. Fig. 2 in [10]

We examined the dose-dependent gene expression changes in candidate marker genes from our previous studies in mouse liver treated with two *N*-nitroso genotoxic hepatocarcinogens to extract key genes, and reported the results of 51 genes determined by qPCR [10]. DEN at doses of 3, 9, 27, and 80 mg/kg body weight (bw) (LD_50_: 200 mg/kg bw, oral) or ENU at doses of 6, 17, 50, and 150 mg/kg bw (LD_50_: 200 mg/kg bw, intraperitoneally) were administered to groups of five 9-week-old B6C3F1 male mice, and the livers were dissected after 4 h and 28 days. Control mice received sterile water. The cDNA was prepared with total RNA from pooled livers and qPCR relative quantitative values were normalized using the *Gapdh* housekeeping gene. A total of 32 genes exhibited a dose response either via increased or decreased expression at least once at 4 or 48 h by DEN or ENU. At 4 h, as shown in Fig. 1 (Fig. 2 in [10]), 26 genes showed an obvious dose-dependent increase in gene expression by DEN [*Aen* (*Isg20l1*), *Bax*, *Btg2*, *Ccng1*, *Ccng2*, *Cdkn1a*, *Cyp4a10*, *Cyp21a1*, *Fos*, *Gadd45b*, *Gdf15*, *Hmox1*, *Hspb1*, *Hspb2*, *Igfbp1*, *Jun*, *Mbd1*, *Mdm2*, *Myc*, *Net1*, *Plk2*, *Pmm1*, *Ppp1r3c*, *Rad52*, *Rcan1*, and *Tubb4b* (*Tubb2c*)] from over 2-fold to 64-fold. Two genes [*Cyp1a2* and *Glul*] showed a dose-dependent decrease in the DEN-treated sample at 4 h. ENU exhibited similar results except for a few genes (*Fabp5* and *Hist1h1c*), although the increase in gene expression to ENU was generally weaker than to DEN. At 28 days, DEN induced a dose-dependent increase, between 2- and 4-fold, in four genes [*Btg2*, *Cdkn1a*, *Cyp21a1*, and *Gdf15*], and a dose-dependent decrease in *Igfbp1* by less than 0.3-fold. ENU exhibited similar results except for the genes *Casp1*, *Gstk1*, *Hspab1*, and *Ung*. Only *Gdf15* displayed a dose-dependent increase in expression on day 28 for both carcinogens. In addition, gene networks were analyzed using Ingenuity Pathway Analysis (IPA, http://www.ingenuity.com/products/ipa), a web-based software application for the analysis, integration, and interpretation of data derived from ‘omics experiments’ such as our qPCR data. Five gene networks were extracted by IPA: Network 1 consisted of genes related to cancer and cell-cycle arrest, such as *Bax*, *Btg2*, *Ccng1*, *Cdkn1a*, *Gadd45b*, *Gdf15*, *Hspb1*, *Hspb2*, *Mdm2*, *Plk2*, and *Pmm1*; Network 2 comprised cell cycle, DNA replication and recombination, repair, and cell death genes, such as *Ccng2*, *Cyp1a2*, *Cyp4a10*, *Cyp21a1*, *Gdf15, Ppp1r3c*, *Rcan1*, and *Tubb4b* (*Tubb2c*).

**Fig. 2:**
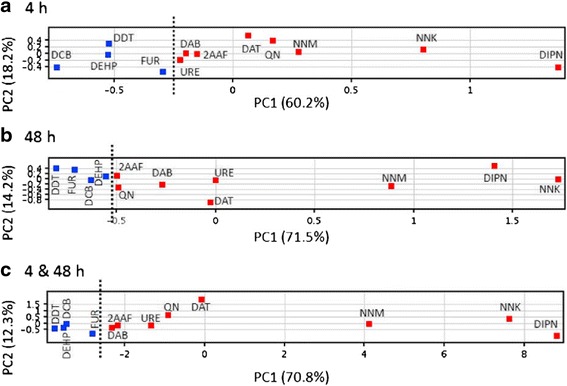
Principal component analysis (PCA) of the gene expression levels between genotoxic and non-genotoxic hepatocarcinogens in mouse liver as quantified by qPCR. The mean values of triplicate qPCR assays for each sample were analyzed statistically using the PCA program in GeneSpringGX11.0.1. The results of the PCA are shown as the two-dimensional contribution scores for component numbers 1 and 2 (PC1 and PC2). The contribution scores were produced by conversion from each eigenvector value, at 4 h with 7 genes (**a**) and at 48 h (**b**) and a combination of both time points (**c**) with 12 genes described in the text. Genotoxic hepatocarcinogens, red colored, DIPN: diisopropanolnitrosamine, NNK: 4-(methylnitrosamino)-1-(3-pyridyl)-1-butanone, NNM: *N*-nitrosomorpholine, QN: quinoline, DAT: 2,4-diaminotoluene, DAB: 4-domethylaninoazobenzene, 2AAF: 2-acetylaminofluorene, URE: urethane) and non-genotoxic hepatocarcinogens (blue-colored, FUR: furan, DDT: dichlorodiphenyltrichloroethane, DEHP: di(2-ethylhezyl)phthalate, DCB: 1,4-dichlorobenzene). Dashed line is added between genotoxic and non-genotoxic hepatocarcinogens. Fig. 2 in [13]

### Time-course changes in gene expression at the acute stage within 48 h

We previously noticed that changes in gene expression were greater at 4 h, while reports on changes in the gene expression profile in rodent liver at the acute stage in the first 48 h after administration of a hepatocarcinogen were limited. We therefore selected key point times at 4 and 48 h from changes in time-dependent gene expression in mouse liver during the acute phase between 4 and 48 h after administration of chrysene, a polycyclic aromatic hydrocarbon (PAH) and genotoxic hepatocarcinogen, as determined by qPCR [11]. Chrysene (100 mg/kg bw) was injected intraperitoneally into groups of three 9-week-old B6C3F1 male mice, and 4, 16, 20, 24, and 48 h later, livers were dissected and processed for gene expression. The cDNA was prepared with total RNA from each individual liver, and the amount of each gene was quantified by qPCR. We reported the results from 50 genes, 35 of which exhibited statistically significant increases at least once within 48 h after exposure to chrysene (Table 1). Fifteen genes [*Bhlhe40*, *Btg2*, *Casp4*, *Ccng2*, *Cdkn1a*, *Crp, Cyp1a1*, *Cyp1a2*, *Fkbp5*, *Gadd45b*, *Gadd45g*, *Hmox1*, *Igfbp1*, *Lcn2*, and *Ly6a*] at 4 h, six genes at 16 h, seven genes at 20 h, seven genes at 24 h, and 10 genes [*Bhlhe40*, *Ccnf*, *Cyp1a1*, *Cyp1a2*, *Ephx1*, *Hhex*, *Hmox1*, *Rcan1*, *Tubb2a*, and *Tubb4b*] at 48 h showed statistically significant increases of more than 2-fold. No significant decreases in gene expression were observed in this study. IPA at 4 h revealed that 7 genes [*Btg2*, *Ccng2*, *Cdkn1a*, *Gadd45b*, *Gadd45g*, *Phlda3*, and *Mdm2*] of 18 genes, which showed statistically significant increases, were associated with cancer, cell cycle, cell death and survival, and cellular growth and proliferation. The expression-increased genes from 16 to 48 h were associated with various biological processes including cancer. *Cyp1a1* and *Cyp1a2* showed remarkably consistent increases in gene expression during 4–48 h. These two genes are associated with toxin metabolism, the oxidation-reduction process, and the induction by carcinogenic polycyclic aromatic hydrocarbons as reported previously [14]. We noticed that the greatest characteristic differences between 4 and 48 h were with 11 genes [*Ly6a*, *Gadd45g*, *Igfbp1*, *Lcn2*, *Casp4*, *Cdkn1a*, *Btg2*, *Ccng2*, *Fkbp5*, *Crp*, and *Gadd45b*], which differentially exhibited a statistically significant increase more than 2-fold at 4 h, and six genes [*Tubb2a*, *Ephx1*, *Hhex*, *Ccnf*, *Rcan1*, and *Tubb4b*] differentially showed a statistically significant increase more than 2-fold at 48 h.

**Table 1:**
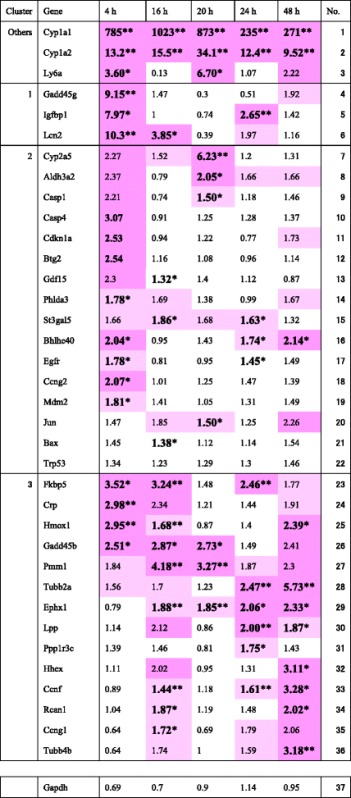
Gene expression ratio (Exp/Cont) and Welch’s *t*-test after chrysene administration

The total RNA was extracted from the individual liver and used to prepare the cDNA. The expression of the 37 genes was quantified by qPCR, and the gene expression ratio (exp/cont) of each gene was calculated. The results were analyzed by Welch’s *t*-test (boldface with ** indicates significant at *P* < 0.01; boldface with * indicates significant at *P* < 0.05). The clusters in Table 1 were sorted through unsupervised hierarchical clustering. The dark pink color shows the values that are higher than 2, and the light pink color shows the values that are higher than 1.5. The table is simplified from Table 3 in [11]

### Discrimination of genotoxic and non-genotoxic hepatocarcinogens at 4 and 48 h in mouse liver

We next successfully showed the discrimination of eight genotoxic hepatocarcinogens from four non-genotoxic hepatocarcinogens at 4 and 48 h in mouse liver by qPCR and statistical analysis using the Dunnett’s test, Welch’s *t*-test, and PCA [12]. Eight genotoxic hepatocarcinogens, 2-acetylaminofuluorene (300 mg/kg bw), 2,4-diaminotoluene (200 mg/kg bw), diisopropanolnitrosamine (500 mg/kg bw), 4-dimethylaminoazobenzene (100 mg/kg bw), 4-(methylnitrosamino)-1-(3-pyridyl)-1-butanone (250 mg/kg bw), *N*-nitrosomorpholine (32 mg/kg bw), quinoline (100 mg/kg bw), and urethane (1000 mg/kg bw) and four non-genotoxic hepatocarcinogens, 1,4-dichlorobenzene (1000 mg/kg bw), dichlorodiphenyltrichloroethane (50 mg/kg bw), DEHP (2000 mg/kg bw), and furan (30 mg/kg bw) were injected intraperitoneally into groups of five 9-week-old B6C3F1 males, livers were collected at 4 and 48 h later, and processed for gene expression. The cDNA was prepared with total RNA from each individual liver, and the gene expression was quantified by qPCR. Control mice received a solvent diluent, either saline or olive oil. We reported the results from 35 genes: 34 genes [*Aen*, *Bax*, *Bhlhe40*, *Btg2*, *Ccnf*, *Ccng1*, *Cdkn1a*, *Cyp1a2*, *Ddit4*, *Ddit4l*, *Egfr*, *Ephx1*, *Gadd45b*, *Gdf15*, *HistH1*, *Hmox1*, *Hspb1*, *Igfbp1*, *Jun*, *Lrp1*, *Ly6a*, *Mbd1*, *Mdm2*, *Phlda3*, *Plk2*, *Pml*, *Pmm1*, *Ppp1r3c*, *Psma3*, *Rad52*, *Rcan1*, *St3gal5*, *Trp53*, and *Tubb4b* (*Tubb2c*)] showed statistically significant changes in their gene expression, at least once at 4 h and/or 48 h, as computed by the Dunnett’s test using the *Gapdh* gene to normalize the data. The statistical significance between the genotoxic and non-genotoxic hepatocarcinogens for each gene was assessed by the Welch’s test at 4 and 48 h after chemical administration. Different sets of 17 genes [*Aen*, *Bax*, *Btg2*, *Ccng1*, *Cdkn1a*, *Egfr*, *Gdf15*, *Hist1h1c*, *Jun*, *Lrp1*, *Mbd1*, *Mdm2*, *Phlda3*, *Plk2*, *Pml*, *Ppp1r3c*, and *Tubb4b* (*Tubb2c*)] at 4 h, and 19 genes [*Aen*, *Bax*, *Btg2*, *Ccnf*, *Ccng1*, *Cdkn1a*, *Ddit4l*, *Ephx1*, *Gadd45b*, *Gdf15*, *Lrp1*, *Ly6a*, *Mdm2*, *Phlda3*, *Plk2*, *Pmm1*, *Ppp1r3c*, *St3gal5*, and *Tubb4b* (*Tubb2c*)] at 48 h showed a statistical significance between the genotoxic and non-genotoxic hepatocarcinogens, as analyzed by the Welch’s test.

Fourteen genes [*Aen*, *Bax*, *Cdkn1a*, *Mdmd2*, *Btg2*, *Ccng1*, *Ddit4*, *Gdf15*, *Hist1h1c*, *Hmox1*, *Hspb1*, *Phlda3*, *Plk2*, and *Pm*l] identified in this study have been reported to be directly associated with *Trp53*. Among these, 11 genes [*Aen*, *Bax*, *Btg2*, *Ccng1*, *Cdkn1a*, *Gdf15*, *Hist1h1c*, *Mdm2*, *Phlda3*, *Plk2*, and *Pml*] showed a statistical significance between the genotoxic and non-genotoxic hepatocarcinogens analyzed by the Welch’s *t*-test at 4 and/or 48 h. Seven major biological processes were extracted from the Gene Ontology analysis (Gene Ontology Consortium: geneontology.org), which were apoptosis, cell cycle and proliferation, DNA damage and repair, oncogenes, and tumor suppression. IPA suggested the DNA damage response pathway resulting from signal transduction by a p53-class mediator was likely leading to the induction of apoptosis. Although we did not observe a significant increase more than 2-fold in Trp53 expression, it was reported that after exposure to DNA-damaging agents, and other stress stimuli, p53 protein was stabilized and activated by a series of post-translational modifications that freed it from MDM2, a ubiquitination ligase responsible for its ubiquitination prior to proteasome degradation [15].

Discrimination of the gene expression profile between the genotoxic and nongenotoxic hepatocarcinogens was achieved by statistical analysis using PCA.

### Useful application of PCA on gene expression profile to discriminate genotoxic and non-genotoxic hepatocarcinogens

We performed a statistical analysis using a logarithmic (log_2_) transformation of the data to stabilize the variance. PCA is a classic statistical procedure and is recently increasingly applied to biological data. PCA involves a mathematical procedure that transforms a number of possibly correlated variables into a smaller number of uncorrelated variables called “principal components”. The first principal component (PC1) accounts for as much of the variability in the data as possible, and each succeeding component accounts for as much as of the remaining variability as possible.

The mathematical formula of PC1 (z_1_) for 4 h is presented as the following:

$$ {\mathrm{z}}_{1\ \left(4\mathrm{h}\right)} = {\mathrm{a}}_{11}{\mathrm{x}}_1 + {\mathrm{a}}_{12}{\mathrm{x}}_2 + \hbox{-} \hbox{-} \hbox{-} \hbox{-} + {\mathrm{a}}_{1\mathrm{p}}{\mathrm{x}}_{\mathrm{p},} $$

where a_1p_ is the eigenvector and x is the canonical logarithmic (log_2_)-transformed gene ratios (exp/cont). PCA was performed using the PCA programs in GeneSpringGX11.0.1 (Agilent Technologies, Santa Clara, CA, USA). Initially, PCA was applied to all 34 logarithmic (log_2_) transformed ratios (exp/cont), and subsequently tried with various candidate gene sets until the optimal discrimination was observed. The candidate genes were selected primarily using the Welch’s *t*-test from the results at 4 h, 48 h, and a combination of both times [12]. PCA can be also performed using a free software R (https://cloud.r-project.org/).

We selected specific genes to obtain an optimal separation between genotoxic hepatocarcinogens and non-genotoxic hepatocarcinogens using PCA. Seven genes [*Btg2*, *Ccnf*, *Ccng1*, *Lrp1*, *Mbd1*, *Phlda3*, and *Tubb4b* (*Tubb2c*)] were used for PCA at 4 h, 12 genes [*Aen*, *Bax*, *Btg2*, *Ccnf*, *Ccng1*, *Cdkn1a*, *Gdf15*, *Lrp1*, *Mbd1*, *Phlda3*, *Plk2*, and *Tubb4b*] at 48 h, and a combination of both time points (Fig. 2).

### Differentiation between genotoxic and non-genotoxic hepatocarcinogens at 4 and 48 h in rat liver

**Fig. 3:**
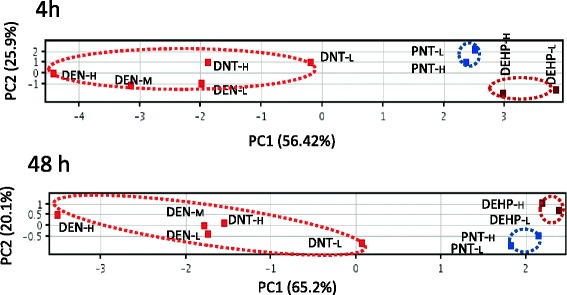
PCA of the gene expression levels under treatment with 3 types of carcinogens in rat liver as quantified by qPCR. Genotoxic hepatocarcinogens (red-colored, DENL: DEN low dose, DEN-M: DEN middle dose, DEN-H: DEN high dose, DNT-L: DNT low dose, DNT-H: DNT high dose), a non-genotoxic carcinogen (brown-colored, DEHP-L: DEHP low dose, DEHP-H: DEHP high dose) and a non-genotoxic non-hepatocarcinogen (blue-colored, PNT-L: PNT low dose, PNT-H: PNT high dose). The mean values of triplicate qPCR assays for each sample were analyzed statistically using the PCA program in GeneSpringGX11.0.1. The results of the PCA are shown as the two-dimensional contribution scores for component numbers 1 and 2 (PC1 and PC2). The contribution scores were produced by conversion from each eigenvector value, at 4 h with 16 genes and at 48 h with 10 genes described in the text. PCA successfully differentiated the genotoxic hepatocarcinogen (*red circle*) from the non-genotoxic hepatocarcinogen (brown circle) and non-genotoxic and non-hepatocarcinogen (*blue circle*) with PC1 and PC2. Fig. 2 in [15]

**Fig. 4:**
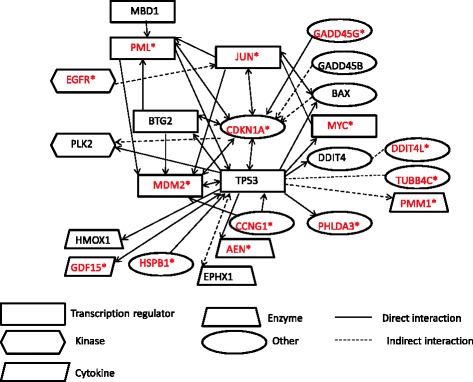
The gene networks and pathways of 24 genes quantified in the present study. The network was constructed from the results of Ingenuity Pathway Analysis, GeneSpring software and references from PubMed. The 15 red-colored genes indicated with an asterisk are genes that significantly contributed to the discrimination of the genotoxic hepatocarcinogens from the non-genotoxic hepatocarcinogen and the non-genotoxic non-hepatocarcinogen by PCA. Fig. 3 in [15]

Finally, we examined hepatocarcinogens in rat liver, and showed successful differentiation of two genotoxic hepatocarcinogens [DEN and 2,6-dinitrotoluene] from a nongenotoxic-hepatocarcinogen [DEHP], and a non-genotoxic non-hepatocarcinogen [phenacetin] at 4 and 48 h by qPCR and PCA [13]. Candidate genes were selected from the data generated in mice. Two genotoxic hepatocarcinogens: DEN (12.5, 25, and 50 mg/kg bw) and 2,6-dinitrotoluene (125 and 250 mg/kb bw), a non-genotoxic hepatocarcinogen: DEHP (1000 and 2000 mg/kg bw), and a non-genotoxic non-hepatocarcinogen: phenacetin (500 and 1000 mg/kg bw) were examined in liver samples from groups of four 4-week-old F344 male mice at 4 and 48 h after a single oral administration of a chemical. Control rats received a solvent of sterile water or olive oil. The cDNA was prepared with total RNA from each individual liver. We reported results from 33 genes: 32 genes [*Aen*, *Bax*, *Btg2*, *Ccnf*, *Ccng1*, *Cdkn1a*, *Cyp21a1*, *Cyp4a1*, *Ddit4l*, *Egfr*, *Ephx1*, *Gadd45b*, *Gadd45g*, *Gdf15*, *Hhex*, *Hmox1*, *Hspb1*, *Igfbp1*, *Jun*, *Lpp*, *Ly6al*, *Mdm2*, *Myc*, *Net1, Phlda3*, *Plk2*, *Pml*, *Pmm1*, *Rcan1*, *Tnf*, *Tp53*, and *Tubb4b* (*Tubb2c*)] exhibited statistically significant changes in expression according to statistical analysis using the Williams’ test and the Dunnett’s test; and a normalized gene, *Gapdh*. The changes appeared to be greater at 4 h than at 48 h. Statistical analysis via PCA successfully differentiated the genotoxic hepatocarcinogens from the nongenotoxic hepatocarcinogen and non-genotoxic non-hepatocarcinogen at 4 h based on 16 genes [*Ccnf*, *Ccng1*, *Cy4a10*, *Ddit4l*, *Egfr*, *Gadd45g*, *Gdf15*, *Hspb1*, *Igfbp1*, *Jun*, *Myc*, *Net1*, *Phlda3*, *Pml*, *Rcan1*, and *Tubb4b* (*Tubb2c*)], and 48 h based on 10 genes [*Aen*, *Ccng1, Cdkn1a*, *Cyp21a1*, *Cyp4a10*, *Gdf15*, *Igfbp1*, *Mdm2*, *Phlda3*, and *Pmm1*] (Fig. 3). Eight major biological processes were extracted from a Gene Ontology analysis: apoptosis, cell cycle and proliferation, DNA damage and repair, oxidative stress, oncogenes, and tumor suppression. IPA suggested the DNA damage response, which signals through a Tp53-mediated pathway and leads to the induction of apoptosis: 24 genes are associated with Tp53 directly or indirectly (Fig. 4). This study showed that mouse candidate marker genes are applicable to rats for the differentiation of the genotoxic hepatocarcinogens from the non-genotoxic hepatocarcinogens examined in this study.

## Discussion

Recently, a new toxicogenomics tool for hepatocarcinogenicity evaluation of drug candidates in rodents (mainly rats) was reported: ToxDBScan (http://www.ra.cs.uni-tuebingen.de/software/ToxDBScan/) [16], which is a web tool offering a quick and easy similarity screening of new drug candidates against two large-scale public databases, which contain expression profiles for substances with known carcinogenic profiles: TG-GATEs (http://toxico.nibiohn.go.jp/english/) [17] and DrugMatrix (https://ntp.niehs.nih.gov/drugmatrix/) [18]. TG-GATEs contains DNA microarray data on 170 chemicals, primarily medicinal compounds. DrugMatrix contains toxicogenomic profiles (DNA microarray data) for 638 different compounds. These compounds include US Food and Drug Administration-approved drugs, drugs approved in Europe and Japan, withdrawn drugs, drugs in preclinical and clinical studies, biochemical standards, and industrial and environmental toxicants. Although these large databases based on DNA microarrays were prepared, the number of published papers on toxicogenomics by DNA microarrays and qPCR in rodent liver or liver cells was not as expected.

Since its first application to toxicogenomics in 2003, PCA is a classic statistical technique that is recently increasingly applied to biological data. Previously, we successfully applied PCA to human lung cancer cell lines [19, 20]. Successful discrimination was performed in some toxicogenomics studies, such as hepatocarcinogens against non-carcinogens in rat liver [21], and carcinogenic PAHs against non-carcinogenic PAHs in HepG2 cells [22]. However, the number of publications using PCA in toxicogenomics is still limited. We are now trying to apply this type of analysis on selected key genes to rodent liver gene expression profiles that have been described previously (unpublished).

Additionally, the involvement of next-generation sequencing (NGS) technology for the study of toxicogenomics is now being introduced [23–25]. Jiang et al. reported that NGS technologies, in comparison to microarray-based technologies, may overcome the current limitations, and are promising for the development of predictive models in the near future [23]. Maslov et al. [24] suggested that the NGS era is well underway; new methods have been developed to directly analyze genetic material in a genome-wide manner with single nucleotide resolution. Moreover, there is no dependency on any particular gene or cell line, and the genetic material derived from any cell or tissue can be analyzed. This makes NGS-based mutagenicity assays particularly suitable for use in genetic toxicology. As toxicology continues to develop, we expect that testing methods will continue to change in concert with increased knowledge and understanding.

## Conclusions

In the present review, we summarize our toxicogenomics collaborative studies. We selected and quantified by qPCR candidate marker genes to discriminate mouse genotoxic hepatocarcinogens from non-genotoxic hepatocarcinogens examined by DNA microarrays. We determined 30 key genes by dose responses in mouse liver gene expression induced by DEN and ENU at 4 h and 28 days, and extracted key times between 4 and 48 h from time-course studies during the acute phase induced by chrysene. Finally, we successfully showed the discrimination in mouse liver of eight genotoxic hepatocarcinogens [2-acetylaminofuluorene, 2,4-diaminotoluene, diisopropanolnitrosamine, 4-dimethylaminoazobenzene, 4-(methylnitrosamino)-1-(3-pyridyl)-1-butanone, *N*-nitrosomorpholine, quinoline, and urethane] from four non-genotoxic hepatocarcinogens [1,4-dichlorobenzene, dichlorodiphenyltrichloroethane, DEHP, and furan] and in rat liver two genotoxic hepatocarcinogens [diethylnitrosamine and 2,6-dinitrotoluene] from a non-genotoxic hepatocarcinogen [DEHP] and a non-genotoxic and non-hepatocarcinogen [phenacetin] determined by qPCR and PCA at 4 and 48 h after administration of chemicals. The subsequent gene pathway studies extracted the DNA damage response, resulting from signal transduction by a p53-class mediator leading to the induction of apoptosis. These studies suggest that application of PCA in the study of toxicogenomics is useful to discriminate genotoxic hepatocarcinogens from non-genotoxic hepatocarcinogens and/or non-hepatocarcinogens in rodent liver.

## Ethical approval

All animal experiments in the original papers [9–13] were conducted in accordance with the NIH Guide for Care and Use of Laboratory Animals and approved by the Animal Care and Use Committee at the Biosafety Research Center, Foods, Drugs, and Pesticides (applicant: NM, RD-07-005; approval: No. 07–066, in 2007) and the Animal Care and Use Committee of the Mitsubishi Chemical Medience Corp (applicant: HS; approval: 2007–0138).

## Abbreviations

DEHP: di(2-ethylhexyl)phthalate
DEN: diethylnitrosamine
ENU: ethylnitrosourea
IPA: ingenuity pathway analysis
JEMS: the Japanese environmental mutagen society
MMS: mammalian mutagenicity study group
NGS: next generation sequencing
PAH: polycyclic aromatic hydrocarbon
PCA: principal component analysis
qPCR: quantitative real-time PCR
